# Prognostic value and immune infiltration analysis of a novel lactylation-related gene signature in endometrial cancer

**DOI:** 10.1016/j.bbrep.2025.102056

**Published:** 2025-05-26

**Authors:** Liqin Gu, Chunnian Zhang, Minjuan Xu, Fang Peng, Ruo-Hui Huang, Deping Luo

**Affiliations:** a Department of Gynecology, Ganzhou People’s Hospital, No. 16 Meiguan Avenue, Zhanggong District, Ganzhou City, Jiangxi Province 341000, China; b Department of Urology, First Affiliated Hospital of Gannan Medical University, No. 128 Jinling West Road, Zhanggong District, Ganzhou City, Jiangxi Province 341000, China; c Gynecological Oncology, Ganzhou Cancer Hospital, No. 19 Huayuanqian Road, Shuidong Town, Zhanggong District, Ganzhou City, Jiangxi Province 341000, China

**Keywords:** Endometrial cancer, Lactylation, Risk model, Immune microenvironment, Tumor mutation burden

## Abstract

**Background:**

Lactylation has been implicated in tumor growth, proliferation, and metastasis; however, its precise relationship with cancer remains poorly understood. This study aims to elucidate the role of lactylation-related genes (LRGs) in the development of endometrial cancer (EC).

**Methods:**

We utilized data from The Cancer Genome Atlas (TCGA) database to analyze the expression and mutation patterns of LRGs in EC. Univariate Cox regression analysis and Lasso-Cox regression analysis were employed to identify prognosis-related genes and construct a risk model. EC samples were stratified into high-risk and low-risk groups based on the risk values derived from the model. These groups were validated using both training and validation cohorts. Additionally, we assessed differences in the immune microenvironment, tumor mutation burden (TMB), and drug response between the high-risk and low-risk groups.

**Results:**

Differentially expressed genes (DEGs) between EC and control samples were identified, and their intersection with LRGs yielded differentially expressed lactylation-related genes (DLRGs). A total of six prognostic DLRGs (PFKM, H3C1, SIRT3, VIM, WAS, and LSP1) were selected and used to construct an EC risk model. Significant differences in prognosis, immune microenvironment, TMB, and drug sensitivity were observed between the high-expression and low-expression groups.

**Conclusion:**

LRGs play a significant role in endometrial cancer by influencing cell growth, the immune microenvironment, and drug response. The six DLRGs included in the risk model may serve as potential prognostic markers and therapeutic targets for EC.

## Highlights

• Identified six lactylation-related genes as potential prognostic markers and therapeutic targets.
• The constructed risk model effectively categorizes endometrial cancer patients into high and low-risk groups.
• Lactylation-related genes influence the immune microenvironment and tumor mutation burden.

## Teaser

Research has shown that lactylation is closely associated with tumor cell growth, proliferation, and metastasis. However, the specific mechanisms linking lactylation to cancer progression remain unclear. To elucidate this relationship, a study was conducted using data from The Cancer Genome Atlas (TCGA) database. The study analyzed the expression and mutation patterns of lactylation-related genes (LRGs) in endometrial cancer (EC). Through single-factor and Lasso-Cox regression analyses, six prognostic differentially expressed LRGs (PFKM, H3C1, SIRT3, VIM, WAS, and LSP1) were identified. These genes were used to construct a risk model that effectively segregated EC samples into high-risk and low-risk categories. Validation using both training and validation sets demonstrated significant differences between the two groups in terms of prognosis, immune microenvironment, tumor mutation burden (TMB), and drug sensitivity. These findings suggest that LRGs play a critical role in EC by influencing cell growth, the immune microenvironment, and drug response. Therefore, these six differentially expressed LRGs could potentially serve as prognostic markers and therapeutic targets for endometrial cancer.

## 1 Introduction

In recent years, endometrial cancer (EC) has emerged as one of the most common malignant tumors of the female reproductive system, with its incidence on the rise. This trend poses a significant threat to women's health [[1], [2], [3]]. While early-stage EC patients generally have a favorable prognosis, those with late-stage disease face a poorer outlook. Despite advancements in treatment and diagnostic technologies, several challenges remain, including difficulties in early diagnosis and the poor prognosis associated with advanced disease [4]. With the rapid development of bioinformatics, an increasing number of studies have begun to focus on genomic, transcriptomic, and epigenetic changes in EC. These efforts aim to deepen our understanding of its pathogenesis and develop personalized treatment strategies.

Lactylation is an emerging protein post-translational modification that was first reported in 2019. This modification involves the binding of lactate molecules to lysine residues and has been detected in various human tissues and cancer types, generating considerable interest in the scientific community [[5], [6], [7]]. Studies have shown that lactylation plays a crucial role in several biological processes, including glycolysis-related cellular functions and the regulation of tumor proliferation [8,9]. For instance, in colorectal cancer, lactylation promotes the expression of the autophagy-enhancing protein RUBCNL, thereby activating the autophagy process. This enhances cancer cell survival and proliferation under hypoxic conditions and leads to resistance to bevacizumab treatment [10]. In gastric cancer, lactylation modifies the K229 site of the METTL16 protein, enhancing its m6A methyltransferase activity. This promotes the stability of FDX1 mRNA and upregulates FDX1 protein expression, ultimately inducing cuproptosis and providing a potential therapeutic target [11]. In bladder cancer, lactylation upregulates the expression of key transcription factors YBX1 and YY1, activating the transcription of their target genes and thereby enhancing cisplatin resistance in cancer cells [12]. These findings highlight the potential importance of lactylation in cancer biology, particularly in the context of cancer metabolism reprogramming and immune regulation. However, the role of lactylation in the development of EC remains unclear.

This study aims to explore the molecular characteristics, tumor progression mechanisms, and potential therapeutic targets of EC using bioinformatics methods. Specifically, various bioinformatics analyses are conducted based on the transcriptome, copy number variation (CNV), tumor mutational burden (TMB), and clinical data of EC samples. A risk model is constructed based on prognosis-related lactylation-related genes (LRGs), and the differences between the high and low-risk groups divided by the model are explored in terms of prognosis, immune microenvironment, drug sensitivity, and other aspects.

## 2 Method

### 2.1 Data acquisition

In this study, transcriptome and tumor mutational burden (TMB) data from 539 EC samples and 35 normal samples in the TCGA-UCEC cohort were collected from The Cancer Genome Atlas (TCGA) database (available at GDC Data Portal Homepage). Corresponding clinical data for the TCGA-UCEC cohort were downloaded from the UCSC Xena database (available at UCSC Xena). Copy number variation (CNV) data were also obtained from the UCSC Xena database. Patients with a survival time of less than 30 days were excluded from the survival analysis. Additionally, a list of 337 lactylation-related genes (LRGs) was compiled from previous literature [[13], [14], [15]]. This list is provided in the supplementary material file "LRGs.txt." The EC samples from the TCGA-UCEC cohort were divided into training and testing sets. The training set comprised 35 normal samples and 268 diseased samples, while the testing set included 271 diseased samples.

### 2.2 Identification method of DLRGs

In this study, differential expression analysis of the transcriptome data from the TCGA database was conducted using the "limma" package in R software. The obtained differentially expressed genes (DEGs) were intersected with the previously collected lactylation-related genes (LRGs) to obtain differential lactylation-related genes (DLRGs). Univariate Cox regression analysis was performed to screen the expression and clinical information data of EC samples, identifying DLRGs significantly associated with prognosis (p < 0.02). Subsequently, the GeneMANIA database (http://genemania.org/) was utilized to identify the interaction relationships among DLRGs and construct a protein-protein interaction (PPI) network. Finally, based on the somatic mutation data in the Mutation Annotation Format (MAF) downloaded from the TCGA database, integrated analysis was conducted using the "Maftools" package in R software [16].

### 2.3 Construction and validation method of the risk model

The lasso-cox algorithm was implemented using the "glmnet" package in R software to further screen genes from DLRGs and select the most suitable genes to construct the prognostic model. A 10-fold cross-validation was conducted to determine the optimal λ value (minimum partial likelihood deviance criterion, lambda.min), which balanced model goodness-of-fit and overfitting risks. Based on the regression coefficients derived from LASSO-Cox and the gene expression levels, we calculated a risk score for each patient. Finally, the prognostic score for cancer research was calculated using the formula based on the selected genes, expressed as follows.

$$Riskscore=\sum_{i=1}^{q}x_{i}\times \beta_{i}$$

Where $x_{i}$ represents the expression data of prognostic DLRGs, q denotes the number of expressed genes, β represents the normalized Cox regression coefficients, and the prognostic score is calculated as the sum of the products of each variable and its corresponding coefficient. All samples were divided into high and low-risk groups based on the median value of the risk score. To validate the significant differences in prognosis between the two groups, Kaplan-Meier (KM) curve analysis was performed using the "survival" and "survminer" packages in R software to assess the prognostic value of EC patients in different risk groups. This study also compared differences in clinical features such as age, T stage, and N stage among these patients. Additionally, receiver operating characteristic (ROC) curves were plotted using the "timeROC" package in R software to evaluate the sensitivity and specificity of the risk model.

### 2.4 Gene Set Enrichment Analysis

Gene Set Enrichment Analysis (GSEA) was conducted on the high and low-risk groups using the "clusterProfiler" package in R software. The gene set utilized was c2.cp.kegg.v7.4.symbols.gmt, with a threshold for pathway selection set at p < 0.05.

### 2.5 Exploration of immune landscape

The immune microenvironment of the high and low-risk groups was evaluated based on the CIBERSORT algorithm, a machine learning algorithm used for high-throughput characterization of different cell types [17]. The following R packages were employed: "preprocessCore" (v1.52.1), "e1071" (v1.7.9), "limma", "ggpubr", "vioplot" (v0.4.0), "ggExtra" (v0.10.0), and "reshape2" (v1.4.4). Additionally, differences in the expression of immune checkpoint loci and HLA genes between the two groups were assessed.

### 2.6 Drug sensitivity analysis

To identify compounds suitable for treating EC patients, the "oncoppredict" package in R software was used to compute drug sensitivity data (IC50 values) for each EC sample. A statistically significant difference in IC50 values between the high and low-risk groups was considered when p < 5e-15.

### 2.7 Statistical analysis

Statistical analyses in this study were conducted using R software (v4.2.0). Differential expression analysis, KM survival curves, univariate Cox regression analysis, and multiple-factor Cox regression analyses were performed using the Wilcoxon test, with p < 0.05 considered statistically significant.

## 3 Results

### 3.1 Identification and analysis of DLRGs

**Fig. 1:**
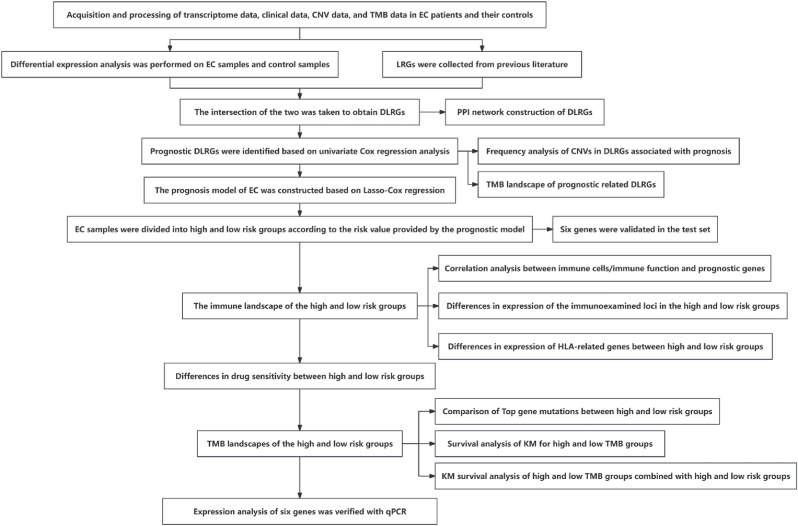
Schematic representation of the workflow in this study.

**Fig. 2:**
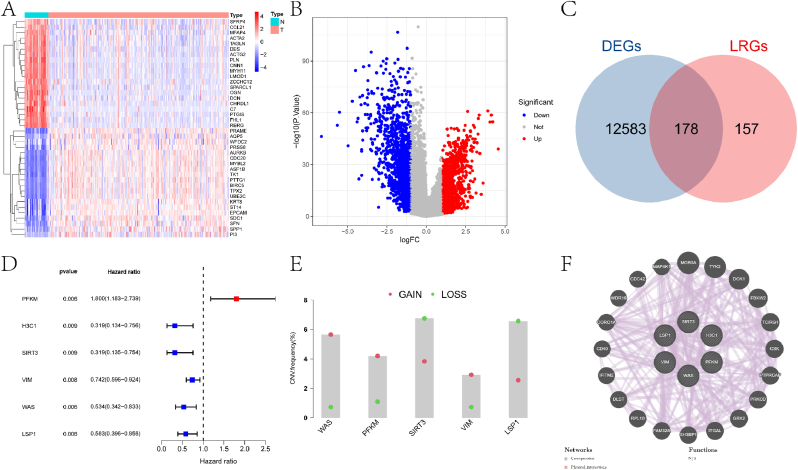
Identification process of DLRGs. (A) Heatmap illustrating the top 20 significantly DEGs identified through differential expression analysis in the control and diseased groups of the TCGA-UCEC cohort. The heatmap displays the logFC values of gene expression levels, with red indicating upregulation and blue indicating downregulation. The color scale ranges from −4 to 4, representing the logFC values. (B) Volcano plot representing the process of differential analysis between the control and diseased groups. The x-axis shows the logFC of gene expression, while the y-axis represents the -log10(p-value) of the statistical significance. The plot highlights significant DEGs (red and blue dots) that meet the criteria of |logFC| > 1 and p-value <0.05. The majority of genes are shown in gray, indicating no significant difference. (C) Venn diagram illustrating the intersection between DEGs and LRGs. The diagram shows the number of overlapping genes (157) between the two sets, representing the DLRGs identified in this study. The total number of DEGs and LRGs is also indicated. (D) Forest plot obtained from univariate Cox regression analysis of DLRGs. The plot displays the hazard ratios (HR) and their 95 % confidence intervals (CI) for each DLRG. The horizontal lines represent the HR values, with the vertical dashed line indicating HR = 1. Genes with HR < 1 are associated with better prognosis, while those with HR > 1 are associated with worse prognosis. (E) Frequency statistics of gain and loss in CNVs of the six prognosis-related DLRGs. The bar chart shows the percentage of samples with CNV gains (red) and losses (blue) for each gene. The y-axis represents the frequency (%), while the x-axis lists the six genes. (F) PPI network diagram showing the interactions among DLRGs. The nodes represent genes, and the edges indicate interactions between them.

**Fig. 3:**
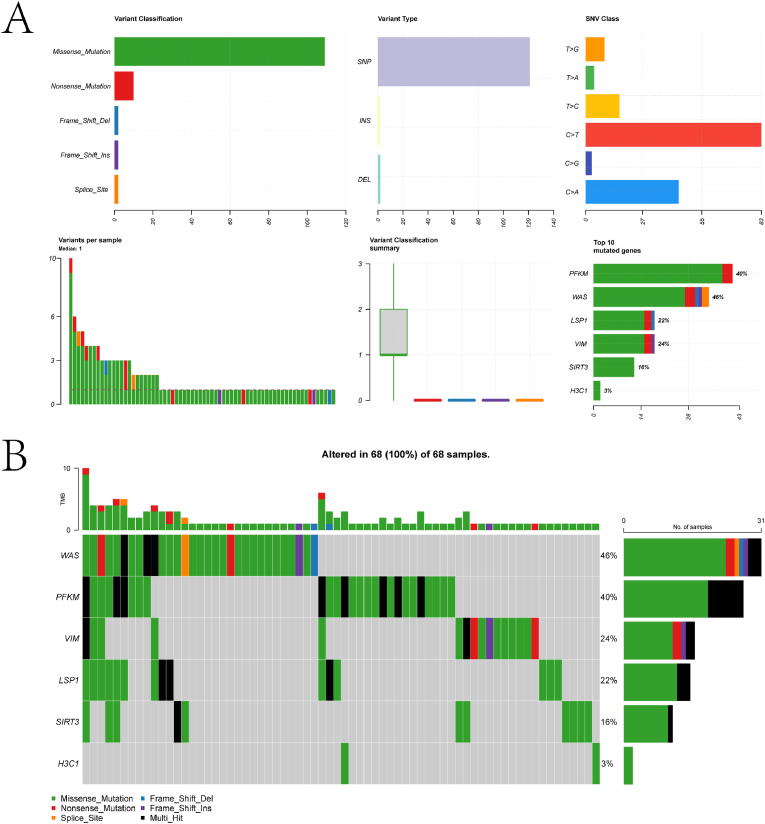
Analysis of Prognostic Gene Structure and Mutation Distribution. (A) Summary plot of MAF analysis obtained using the maftools package. The figure sequentially presents the following information: Variant Classification: Bar chart showing the distribution of different variant classifications (e.g., Missense_Mutation, Nonsense_Mutation, Splice_Site, Frame_Shift_Ins, Frame_Shift_Del) in the dataset. The y-axis represents the count of variants, while the x-axis lists the variant classifications. Variant Type: Bar chart displaying the distribution of variant types (e.g., DEL, INS, SNP). The y-axis represents the count of variants, while the x-axis lists the variant types. SNV Class: Bar chart showing the distribution of single nucleotide variant (SNV) classes (e.g., C > A, C > G, C > T, T > C, T > A, T > G). The y-axis represents the count of variants, while the x-axis lists the SNV classes. Variants per Sample: Histogram showing the number of variants per sample. The x-axis represents the number of variants per sample, while the y-axis shows the frequency of samples with that number of variants. The median number of variants per sample is indicated. Summary of Variants: Bar chart summarizing the variant classification for each of the six prognostic genes (H3C1, SIRT3, VIM, LSP1, WAS, PFKM). The y-axis represents the count of variants, while the x-axis lists the genes. Ranking Information: Bar chart showing the top 10 mutated genes in the dataset. The y-axis represents the percentage of samples with mutations in each gene, while the x-axis lists the genes. The genes are ranked by mutation frequency. (B) Landscape plot of somatic mutations in the TCGA-UCEC cohort. The plot provides a visual summary of the mutation distribution across samples and genes. Each row represents a sample, and each column represents a gene. The presence of a mutation in a gene is indicated by a colored cell, with different colors representing different types of mutations. The plot also includes a summary of the mutation frequency for each gene and the number of samples with mutations.

Fig. 1 illustrates the overall workflow of this study. Differential expression analysis was first conducted on the control and EC samples from the TCGA-UCEC cohort, identifying 12,761 DEGs with a threshold of |logFC| > 0.2 and adjusted p-value <0.05 (Fig. 2A–B). Comprehensive summary information on all genes after differential expression analysis is provided in the supplementary material file "diff_all.xls." Subsequently, intersecting these LRGs yielded 178 differentially expressed LRGs (DLRGs) (Fig. 2C). Univariate Cox regression analysis of these DLRGs revealed that the expression of six genes (PFKM, H3C1, SIRT3, VIM, WAS, and LSP1) was significantly correlated with the prognosis of EC patients (Fig. 2D). Among these, PFKM was identified as a risk factor for EC, while the remaining genes were protective factors. More specific information is provided in the supplementary material file "tcga.uniCox.txt." We further investigated the copy number variations (CNVs) of these six genes to explore how these lactylation-related genes change on chromosomes. The analysis indicated that WAS was the most significantly altered "GAIN" gene, while SIRT3 was the most significantly "LOST" gene (Fig. 2E). Protein-protein interaction (PPI) network analysis revealed that most proteins encoded by DLRGs are closely interconnected in a complex manner (Fig. 2F). Detailed information on PPI network construction is provided in the supplementary material file "PPI.txt." Finally, we summarized and ranked the mutation frequencies of the six genes using the maftools package (Fig. 3A–B). The results showed that WAS had the highest mutation frequency.

### 3.2 Construction and validation results of the risk model

**Fig. 4:**
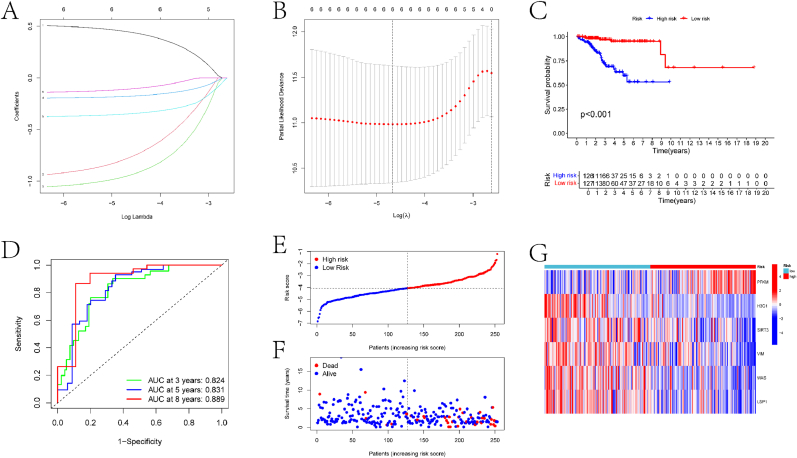
Construction process of the prognostic model based on Lasso-Cox. (A) Lasso regression coefficient plot. This panel illustrates the determination of DLRGs and their corresponding coefficients through Lasso regression analysis. The x-axis represents the log(lambda) value, while the y-axis indicates the coefficient values for each gene. Each line corresponds to a gene, with the coefficients shrinking towards zero as the log(lambda) value increases. (B) Partial likelihood deviance plot for Lasso regression. This panel displays the partial likelihood deviance (y-axis) against the log(lambda) value (x-axis). The vertical dashed line indicates the optimal lambda value chosen for the model, which corresponds to the point where the deviance is minimized. (C) KM survival curves for high and low-risk groups in the training set. The x-axis represents time (in years), and the y-axis represents the survival probability. The plot shows the difference in survival outcomes between the high-risk group (red curve) and the low-risk group (blue curve). The p-value indicates the statistical significance of the difference in survival between the two groups. A lower survival probability in the high-risk group suggests that the risk model effectively stratifies patients based on their prognosis. (D) ROC curves of the risk model predicting the 3-year, 5-year, and 8-year survival rates of EC patients in the training set. The x-axis represents 1-specificity, and the y-axis represents sensitivity. The AUC values for each time point are provided, indicating the model's predictive accuracy. (E) Distribution curve of risk scores in the training set. The x-axis represents the risk score, and the y-axis represents the density of patients. The plot shows the distribution of risk scores among the patients, with a clear separation between high-risk and low-risk groups. (F) Scatter plot of risk scores in the training set. The x-axis represents the patients (ordered by increasing risk score), and the y-axis represents the risk score. The plot displays the risk scores for each patient, with high-risk patients on the right side and low-risk patients on the left side. The survival status (dead or alive) is also indicated for each patient. (G) Heatmap of prognostic gene expression in two groups of EC samples in the training set. The heatmap displays the expression levels of the six prognostic genes in high-risk and low-risk groups. The color scale indicates the expression levels, with red representing high expression and blue representing low expression.

This study further analyzed the six prognostic-related genes to construct a risk model for EC. Specifically, Lasso-Cox regression analysis was utilized to build the risk model using the six prognostic genes from the training set (Fig. 4A–B). The model categorized all EC samples into high-risk and low-risk groups based on their risk scores. Results from the training set indicated significant differences in prognosis between the two risk groups (Fig. 4C). The model achievedAUC values of 0.824, 0.831, and 0.889 for predicting patient survival at 3, 5, and 8 years, respectively (Fig. 4D). Survival analysis and scatter plots demonstrated poorer survival outcomes in the high-risk group (Fig. 4D). The high-risk group contained a higher proportion of deceased samples (Fig. 4E–F), and the expression levels of the prognostic genes were significantly different between the two groups (Fig. 4G).

**Fig. 5:**
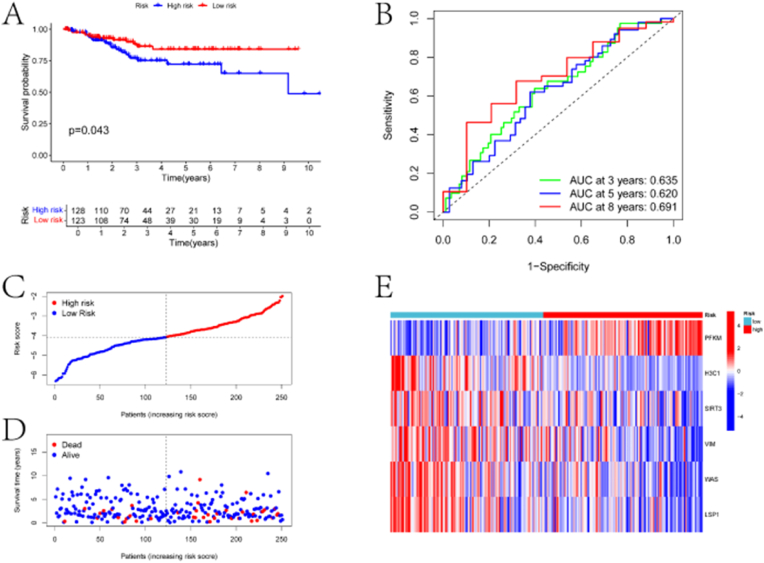
Validation results of the risk model. (A) KM survival curves for high and low-risk groups in the test set. The x-axis represents time (in years), and the y-axis represents the survival probability. The plot shows the difference in survival outcomes between the high-risk group (red curve) and the low-risk group (blue curve). The p-value (p = 0.043) indicates the statistical significance of the difference in survival between the two groups. (B) Receiver Operating Characteristic (ROC) curves of the risk model predicting the 3-year, 5-year, and 8-year survival rates of EC patients in the test set. The x-axis represents 1-specificity (false positive rate), and the y-axis represents sensitivity (true positive rate). The area under the curve (AUC) values for each time point are provided (AUC at 3 years: 0.635, AUC at 5 years: 0.620, AUC at 8 years: 0.691), indicating the model's predictive accuracy. (C) Distribution curve of risk scores in the test set. The x-axis represents the risk score, and the y-axis represents the density of patients. The plot shows the distribution of risk scores among the patients in the test set, with a clear separation between high-risk and low-risk groups. (D) Scatter plot of risk scores in the test set. The x-axis represents the patients (ordered by increasing risk score), and the y-axis represents the risk score. The plot displays the risk scores for each patient, with high-risk patients on the right side and low-risk patients on the left side. The survival status (dead or alive) is also indicated for each patient. (E) Heatmap of prognostic gene expression in two groups of EC samples in the test set. The heatmap displays the expression levels of the prognostic genes in high-risk and low-risk groups. The color scale indicates the expression levels, with red representing high expression and blue representing low expression.

**Fig. 6:**
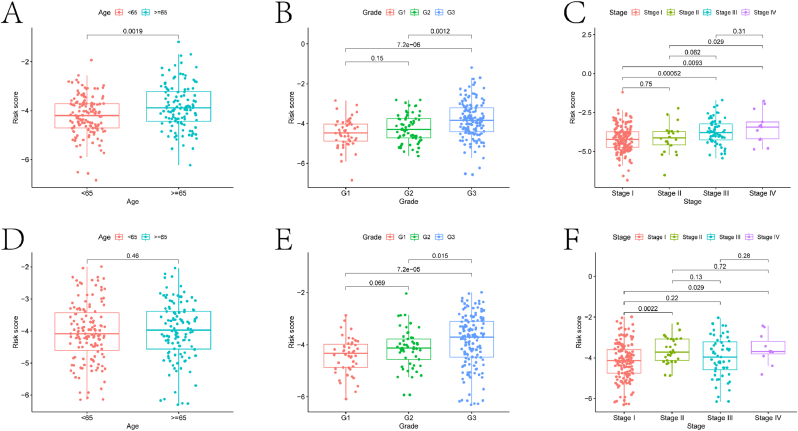
Boxplots of clinical indicators in different risk groups. A-C depict the boxplots of differences in age, Grade classification, and Stage classification among EC samples in the high and low-risk groups in the training set, respectively. D-F represent the boxplots of differences in age, Grade classification, and Stage classification among EC samples in the high and low-risk groups in the test set, respectively. The boxplot illustrates the median, quartiles, and range of stage classification in each risk group.

Subsequently, the model was validated on the testing set. Significant differences in survival were observed between the high-risk and low-risk groups (p < 0.05) (Fig. 5A). ROC curve analysis yielded AUC values of 0.635, 0.620, and 0.691 for predicting survival at 3, 5, and 8 years, respectively (Fig. 5B). Risk score curves and scatter plots indicated a higher proportion of deceased samples in the high-risk group (Fig. 5C–D). Heatmap analysis revealed significant differences in the expression of the six genes between the two groups (Fig. 5E). Additionally, to assess the accuracy of individual prognostic-related DLRGs in predicting patient prognosis, Kaplan-Meier (KM) analysis was conducted using the Ualcan database for the six prognostic-related genes (Supplementary Material, Fig. S1). Finally, differences in clinical factors between the high-risk and low-risk groups were explored. In the training set, significant differences were observed between the two groups in terms of age, G1 and G3 grading, G2 and G3, Stage I and Stage III, and Stage I and Stage IV (Fig. 6A–C). In the testing set, significant differences were observed in G1 and G3 grading, G2 and G3, Stage I and Stage II, and Stage I and Stage IV (Fig. 6D–F).

### 3.3 Construction and validation results of the forest plot model

**Fig. 7:**
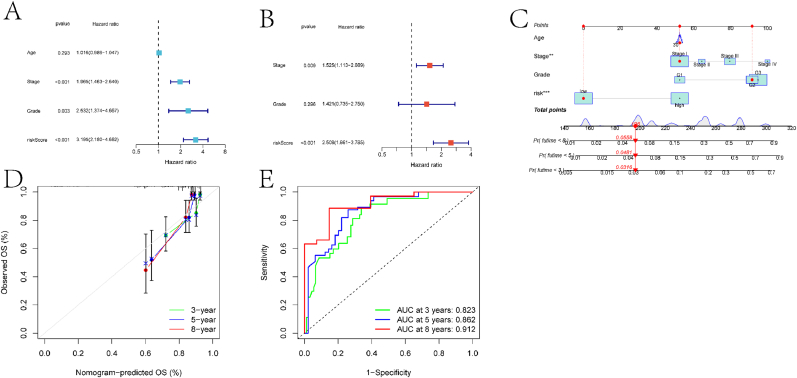
Construction results of the forest plot model. (A) Univariate Cox regression analysis results for clinical characteristics and risk scores in EC patients. HR and p-values are shown. (B) Multivariate Cox regression analysis results for clinical characteristics and risk scores in EC patients. Adjusted HRs and p-values are displayed. (C) Forest plot model constructed based on multivariate Cox regression analysis. HRs and 95 % CI are shown for each variable. (D) Calibration curve of the forest plot model, comparing predicted and observed survival probabilities at 3, 5, and 8 years. € ROC curves of the forest plot model predicting patient survival rates at 3, 5, and 8 years. AUC values are provided for each time point.

In order to explore independent prognostic factors for EC, this study utilized univariate Cox regression to identify age, Stage classification, Grade classification, and risk score as significant factors associated with prognosis (Fig. 7A–B). Furthermore, applying multivariate Cox regression identified Stage classification, Grade classification, and risk score as independent prognostic factors. Based on these factors, a forest plot model was constructed (Fig. 7C–D), which predicted AUCs for patient survival at 3, 5, and 8 years to be 0.823, 0.662, and 0.912, respectively. Compared to the prognostic model, the forest plot model demonstrated higher AUC values (Fig. 7E).

### 3.4 Immune landscape in different risk groups

**Fig. 8:**
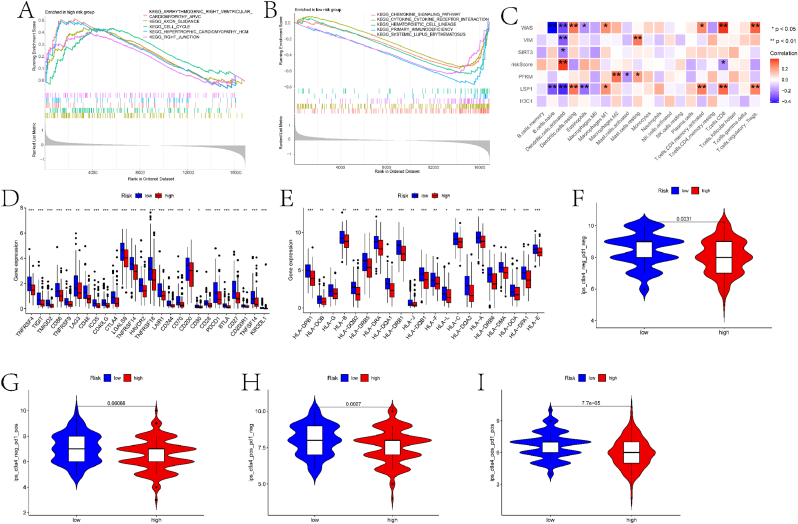
Enrichment analysis and immune landscape of high and low-risk groups. (A) GSEA results for the high-risk group. The plot shows the running enrichment score (y-axis) against the rank in the ordered dataset (x-axis). (B) GSEA results for the low-risk group. The plot shows the running enrichment score (y-axis) against the rank in the ordered dataset (x-axis). (C) Correlation heatmap between immune cells/functions and prognostic genes. The heatmap displays the correlation coefficients (y-axis) between various immune cells and the prognostic genes. (D) Differential expression boxplots of immune checkpoint genes between the high and low-risk groups. The boxplots show the expression levels of immune checkpoint genes in the high-risk (red) and low-risk (blue) groups. (E) Differential expression boxplots of HLA-related genes between the high and low-risk groups. The boxplots show the expression levels of HLA-related genes in the high-risk (red) and low-risk (blue) groups. (F) Risk score distribution and survival analysis for the ips_ctla4_neg_pd1_neg group (no response to CTLA-4 and PD-1 antibodies). (G) Risk score distribution and survival analysis for the ips_ctla4_neg_pd1_pos group (no response to CTLA-4 but response to PD-1). (H) Risk score distribution and survival analysis for the ips_ctla4_pos_pd1_neg group (response to CTLA-4 but no response to PD-1). (I) Risk score distribution and survival analysis for the ips_ctla4_pos_pd1_pos group (response to both CTLA-4 and PD-1 antibodies).

To identify differential functional pathways between high-risk and low-risk groups stratified based on gene signatures, we conducted GSEA using data from the TCGA database (Fig. 8A–B). The specific results are detailed in the supplementary material file "GSEA.result.txt." We will further discuss the roles of these pathways in EC development in the discussion section. Subsequently, we evaluated the correlation between the six prognostic genes and the abundance of immune cell infiltration and immune function scores using the CIBERSORT algorithm (Fig. 8C). Some genes exhibited significant correlations with specific immune cells and immune functions. We also examined the expression of immune checkpoint genes and HLA-related genes in both groups, with the majority of genes showing significant differences in expression between the high-risk and low-risk groups (Fig. 8D–E). The Immune Prognostic Score (IPS) of immunogenicity predicts the potential response of tumor patients to immunotherapy. Therefore, this study assessed the potential response to immunotherapy in both high-risk and low-risk groups by examining the relationship between samples and IPS. Significant differences were observed in all four scenarios for both groups (Fig. 8F–I).

### 3.5 Drug sensitivity analysis and TMB landscape in risk groups

**Fig. 9:**
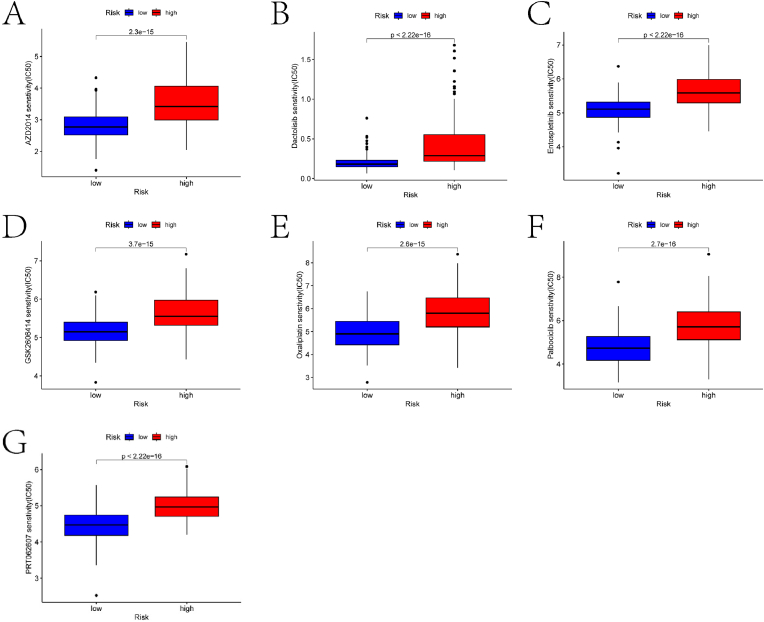
Drug sensitivity analysis results. (A)–(G) Box plots showing the differences in IC50 values for various drugs between the high and low-risk groups. Each panel represents a different drug, with the x-axis indicating the risk group (low risk vs. high risk) and the y-axis representing the IC50 values.

**Fig. 10:**
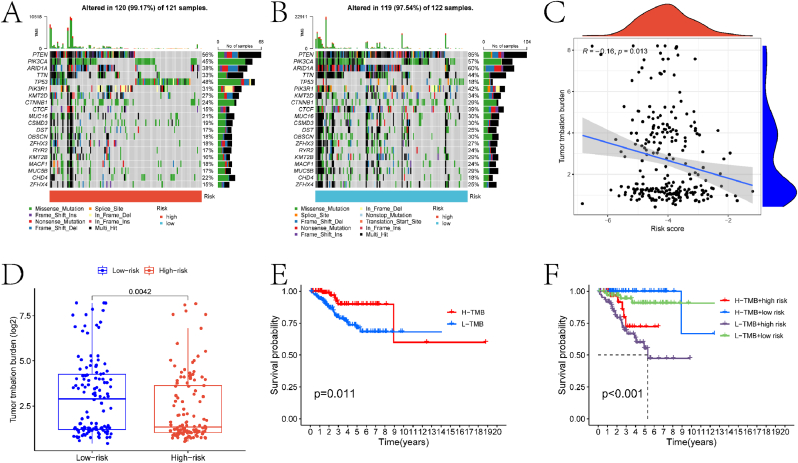
TMB landscape in high and low-risk groups. (A) Waterfall plot showing the top gene mutation profiles in the high-risk group. The x-axis lists the samples, and the y-axis represents the number of mutations. Each bar represents a sample, with different colors indicating different types of mutations (e.g., missense, nonsense, frame shift). (B) Waterfall plot showing the top gene mutation profiles in the low-risk group. (C) Scatter plot illustrating the correlation between risk scores and TMB. The x-axis represents the risk score, and the y-axis represents the TMB (log2-transformed). Each dot represents a sample, with colors indicating risk group (high risk vs. low risk). (D) Box plot indicating the difference in TMB between the high and low-risk groups. The x-axis represents the risk group (high risk vs. low risk), and the y-axis represents the TMB (log2-transformed). (E) KM survival curves for the high and low TMB groups. The x-axis represents time (in years), and the y-axis represents the survival probability. (F) KM survival curves for the combined grouping of high and low TMB and high and low-risk groups. The x-axis represents time (in years), and the y-axis represents the survival probability.

This study identified drugs with significantly different IC50 values between the high-risk and low-risk groups (Fig. 9A–G). The roles of these drugs in EC development and their potential impact on treatment will be discussed in subsequent sections. Additionally, we analyzed the TMB landscape in both groups. Fig. 10A–B shows waterfall plots highlighting the top gene mutation frequencies in the high-risk and low-risk groups, respectively. These plots reveal clear differences in mutation frequencies between the two groups. A significant negative correlation was observed between sample TMB scores and risk scores (Fig. 10C). Based on the TMB scores of the EC samples, we divided the samples into high TMB and low TMB groups. Significant differences were noted in both TMB scores and survival outcomes between these two groups (Fig. 10D–E). Furthermore, survival outcomes also significantly differed among the four groups formed by the combined classification of high and low TMB groups and high and low-risk groups (Fig. 10F).

### 3.6 Validation of prognostic gene expression

**Fig. 11:**
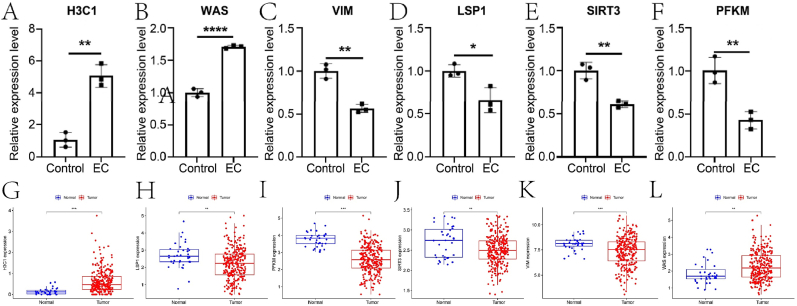
Validation of expression of six prognostic genes. (A)–(F) The validation results of the expression trends of the six genes in both groups were confirmed using qRT-PCR. (G)–(L) The box plots show the expression of the six genes in the control group and EC group.

This study further validated the expression levels of the six prognostic genes. Fig. 11A–F presents box plots illustrating the significant differences in expression of these six genes between the control group and the EC group. Subsequently, we confirmed these expression differences using qRT-PCR (Fig. 11A–F). This is consistent with the sequencing results at the transcriptome level(Fig. 11).

## 4 Discussion

Recent studies have shown that lactate is involved in processes such as tumor cell growth, proliferation, and metastasis. However, its role in the development of EC remains unclear. To address this gap, this study adopted a bioinformatics approach. We first collected and organized EC transcriptome data from the TCGA database. DEGs between the control and EC groups were identified using the limma algorithm. After intersecting these DEGs with previously collected LRGs, univariate Cox regression analysis was performed on the intersecting genes. This process identified six prognostic-related genes: PFKM, H3C1, SIRT3, VIM, WAS, and LSP1. Endometrial cancer is closely related to the regulation of oxidative stress [18]. Liu et al. constructed an oxidative stress-related gene risk model and found an association between the expression level of H3C1 and immune-infiltrating cells in uterine corpus endometrial carcinoma (UCEC) [19]. The Sirtuins (SIRT1-SIRT7) are involved in hormone imbalance, metabolism, and aging, and they profoundly affect EC [20]. SIRT3, a member of the Sirtuin family, encodes a mitochondrial protein that can eliminate reactive oxygen species, inhibit cell apoptosis, and prevent cancer cell formation. It also has profound effects on nuclear gene expression, cancer, cardiovascular diseases, neuroprotection, aging, and metabolic control. The expression of VIM predicts less tumor metastasis and higher overall survival in EC, making it an excellent prognostic biomarker for this cancer [21]. WAS is a protein-coding gene associated with diseases such as Wiskott-Aldrich syndrome and neutropenia, which can lead to malignant tumor development [22]. In addition to identifying these genes, this study also explored their mutational landscape.

To further facilitate stratified patient treatment for endometrial cancer (EC), this study constructed an EC risk model using Lasso-Cox regression analysis. Validation results from both the training and testing sets demonstrated significant differences in prognosis between high-risk and low-risk group samples identified by this model. Additionally, this study identified independent prognostic factors for EC based on the risk model. A columnar line graph model, which exhibited superior predictive performance, was constructed based on these prognostic factors, highlighting its potential clinical utility. Moreover, this study explored differences in KEGG pathways between the high-risk and low-risk groups. For the high-risk group, resveratrol can inhibit EC by activating the mitochondrial-mediated apoptosis pathway and inducing G2/M cell cycle arrest [23]. Tseng et al. reported that the axon guidance molecule Sema3E is highly expressed in human high-grade ovarian endometrioid carcinoma [24]. For the low-risk group, systemic lupus erythematosus is associated with an overall increased risk of cancer [25]. TPX2 affects the malignant progression of endometrial cancer cells by coupling the CX3CR1/CXCL10 chemokine pathway to the PI3K/Akt signaling pathway [26].

Thirdly, to confirm the stratification ability of the risk model, this study explored the immune landscape of the two risk groups. Significant correlations were observed between various immune cells/functions and prognostic-related genes. For example, within the immune cells depicted in Fig. 8C, naive B cells refer to primary B cells that have not undergone maturation and activation processes, exhibiting high plasticity in the immune system. Research has confirmed the significant role of B cells in endometrial cancer (EC) [27]. Additionally, wild-type ARID1A is associated with significantly higher abundance of macrophages and activated dendritic cells in EC patients [28]. Song et al. identified key genes of endometrial cancer through single-cell analysis of macrophages [29]. Subsequently, this study explored the expression differences of immune checkpoint genes and HLA-related genes between the two risk groups. TNFRSF4 emerged as a prominent biomarker for prognosis and immunomodulation in EC [30]. Furthermore, artesunate-induced ATG5-related autophagy enhances the cytotoxicity of NK92 cells against EC cells through interactions between CD155 and CD226/TIGIT [31].

Fourthly, to confirm the differences in drug sensitivity analysis between the high and low-risk groups, this study found significant disparities in the IC50 values of various drugs between the two groups. Clinical trials are currently underway to evaluate the combination therapy of AZD2014 with MEK inhibitors for recurrent metastatic EC (https://classic.clinicaltrials.gov/ct2/show/NCT01011933). Fracasso et al. investigated the phase II efficacy of oxaliplatin as second-line chemotherapy for EC patients [32]. Targeted combination therapy with the FGFR inhibitor lenvatinib and the cell cycle inhibitor palbociclib shows synergistic therapeutic effects on the in vivo growth of dedifferentiated endometrial carcinoma (DEC) [33].

Finally, this study investigated the TMB landscape in different risk groups and its association with prognosis in EC patients. Significant differences were observed in the mutation frequencies of genes between the high-risk and low-risk groups. Additionally, a significant negative correlation was found between TMB scores and risk scores in EC patients. Furthermore, significant differences in survival outcomes were noted among EC samples grouped by TMB score alone and among those grouped by both TMB and risk scores.

While this study provides valuable insights into the role of lactate-related genes in EC prognosis and immune landscape, several limitations should be acknowledged. First, the study relies solely on bioinformatics analysis and publicly available datasets, which may limit the generalizability of the findings to broader patient populations. Second, the lack of experimental validation for the identified prognostic genes and risk model is a significant limitation. Future studies should include in vitro and in vivo experiments to validate the biological functions of these genes and the clinical utility of the risk model. Third, the study did not explore the potential interactions between the identified genes and other molecular pathways involved in EC progression. Further research is needed to elucidate these complex interactions and their implications for targeted therapies.Future research should focus on validating the prognostic value of the identified genes and risk model in independent cohorts. Additionally, functional studies should be conducted to explore the mechanisms by which these genes influence EC development and progression. Investigating the potential therapeutic targets and biomarkers derived from this study in preclinical models and clinical trials is also warranted. Furthermore, exploring the interactions between the identified genes and other molecular pathways, such as the immune system and metabolic pathways, may provide new insights into the pathogenesis of EC and lead to the development of novel therapeutic strategies.

## 5 Conclusion

In summary, this study identified six DLGs associated with the prognosis of EC patients. These genes can be used to categorize patients into two groups with significantly different prognoses, allowing for accurate prediction of their outcomes. Furthermore, we analyzed drugs with significantly different IC50 values between the high-risk and low-risk groups. Lastly, we observed differences between the two groups in terms of the immune microenvironment and tumor mutational burden (TMB). Overall, the findings of this study suggest that incorporating a DLG-based score into clinical practice may serve as a valuable prognostic tool for EC.

## Consent to participate

Not applicable.

## Consent to publish

Not applicable.

## Author contributions statement

Gu Liqin: Conceptualization, methodology, software, formal analysis, investigation, data curation, writing - original draft preparation.

Zhang Chunnian: Methodology, software, formal analysis, investigation, data curation, writing - review & editing, visualization.

Xu Minjuan: Conceptualization, methodology, resources, data curation, writing - review & editing, supervision, project administration.

Peng Fang: Investigation, resources, data curation, writing - review & editing, visualization.

Huang Ruohui: Conceptualization, methodology, resources, writing - review & editing, supervision, project administration, funding acquisition.

Deping Luo (Corresponding Author):Conceptualization, methodology, resources, writing - review & editing, supervision.

## Ethical approval

The utilization of patient tissue samples received approval from the Ethics Committee of the Fourth Hospital of Hebei Medical University (Approval Number: 2024KY008).

## Clinical trial number

Not applicable.

## Funding

This study was supported by the Science and Technology Program Project of Ganzhou Municipal Bureau of Science and Technology, Ganzhou Key Research and Development Program Project (GZ2024YLJ065).

## Declaration of competing interest

The authors declare that they have no competing interests.

## Data Availability

The data that has been used is confidential.
