# Supplementary figures and images for: Prognostic value and immune infiltration analysis of a novel lactylation-related gene signature in endometrial cancer
Source: Biochem Biophys Rep. 2025 May 26;42:102056. doi: 10.1016/j.bbrep.2025.102056 (PMC12159215; doi:10.1016/j.bbrep.2025.102056)

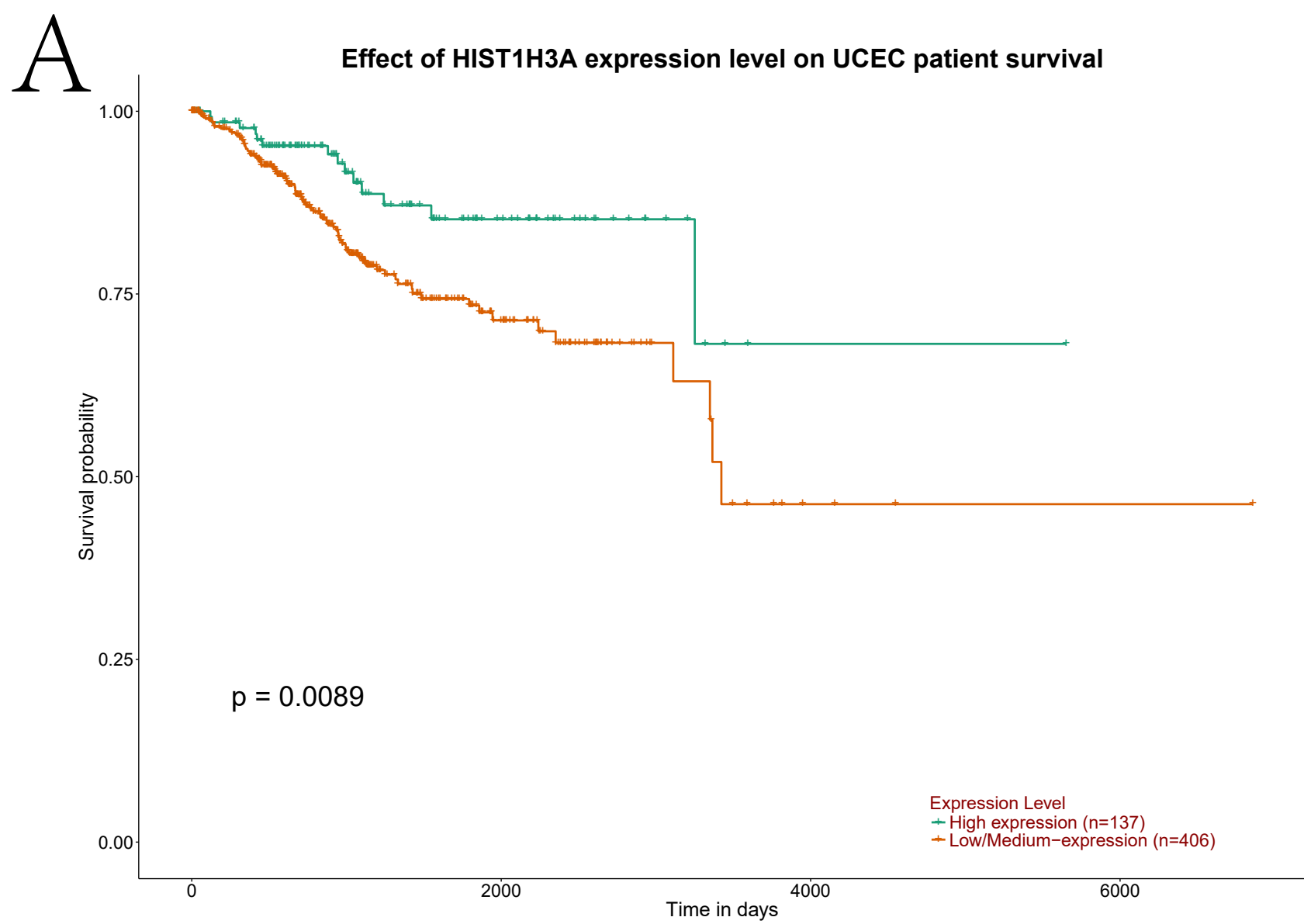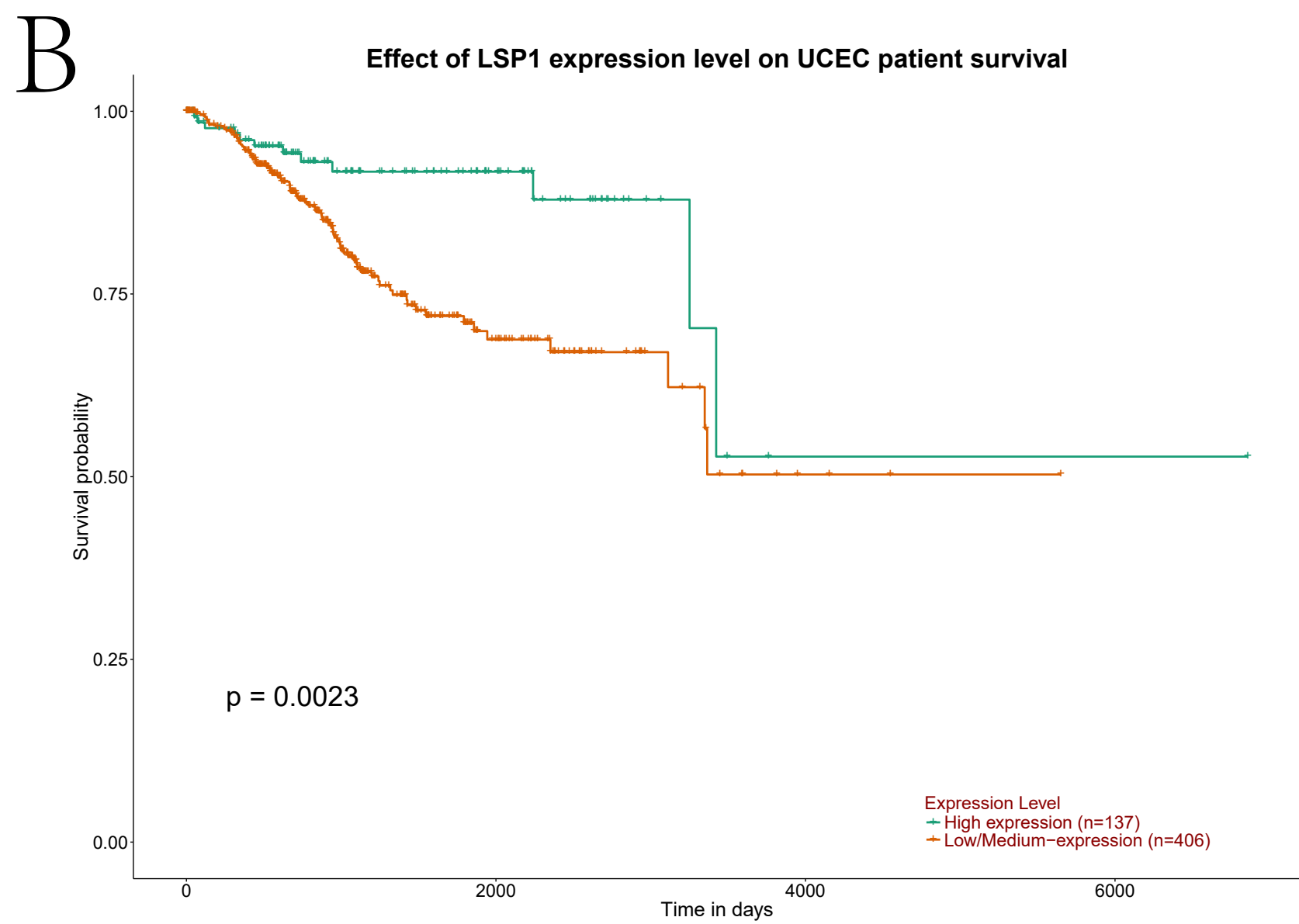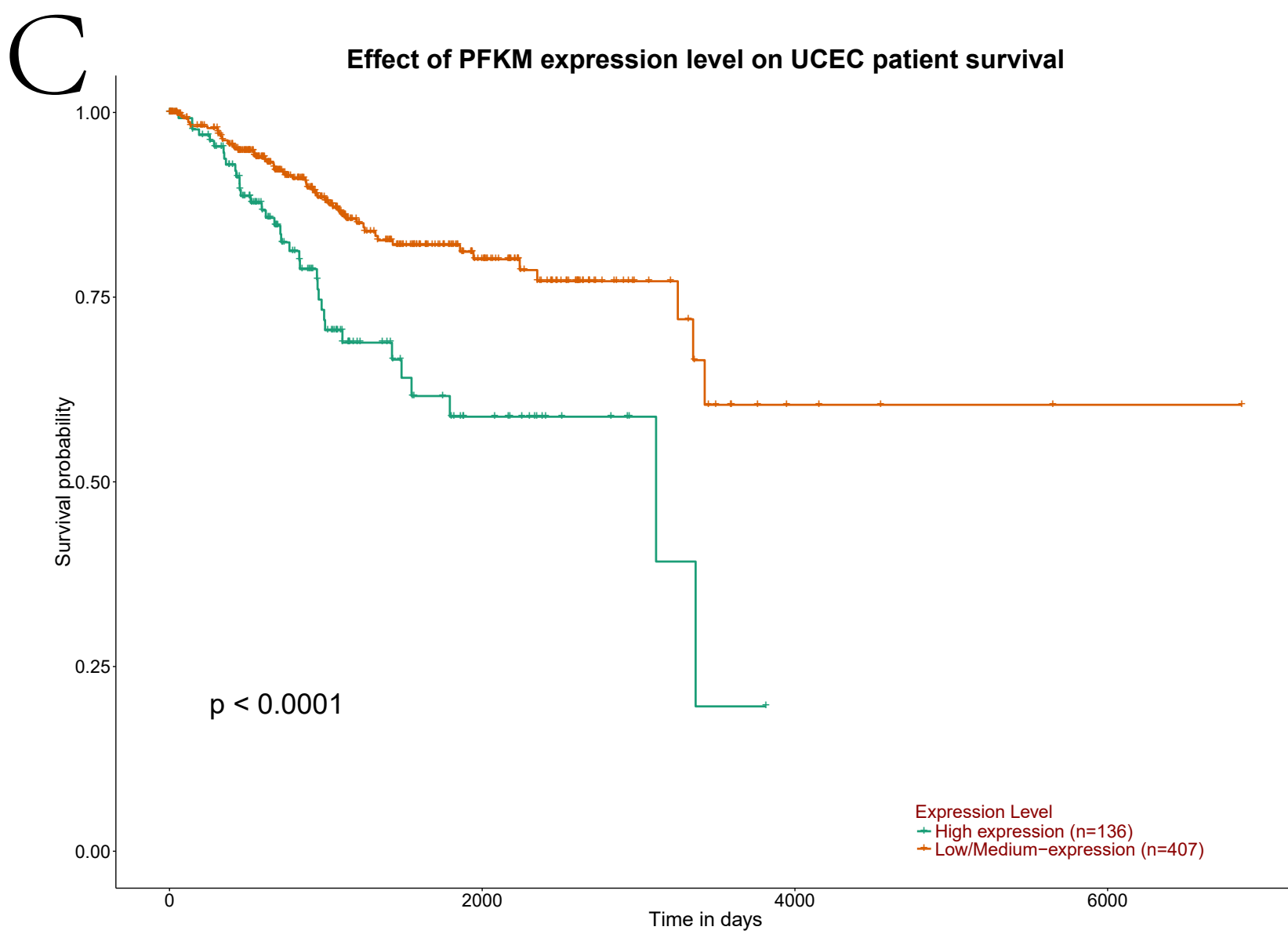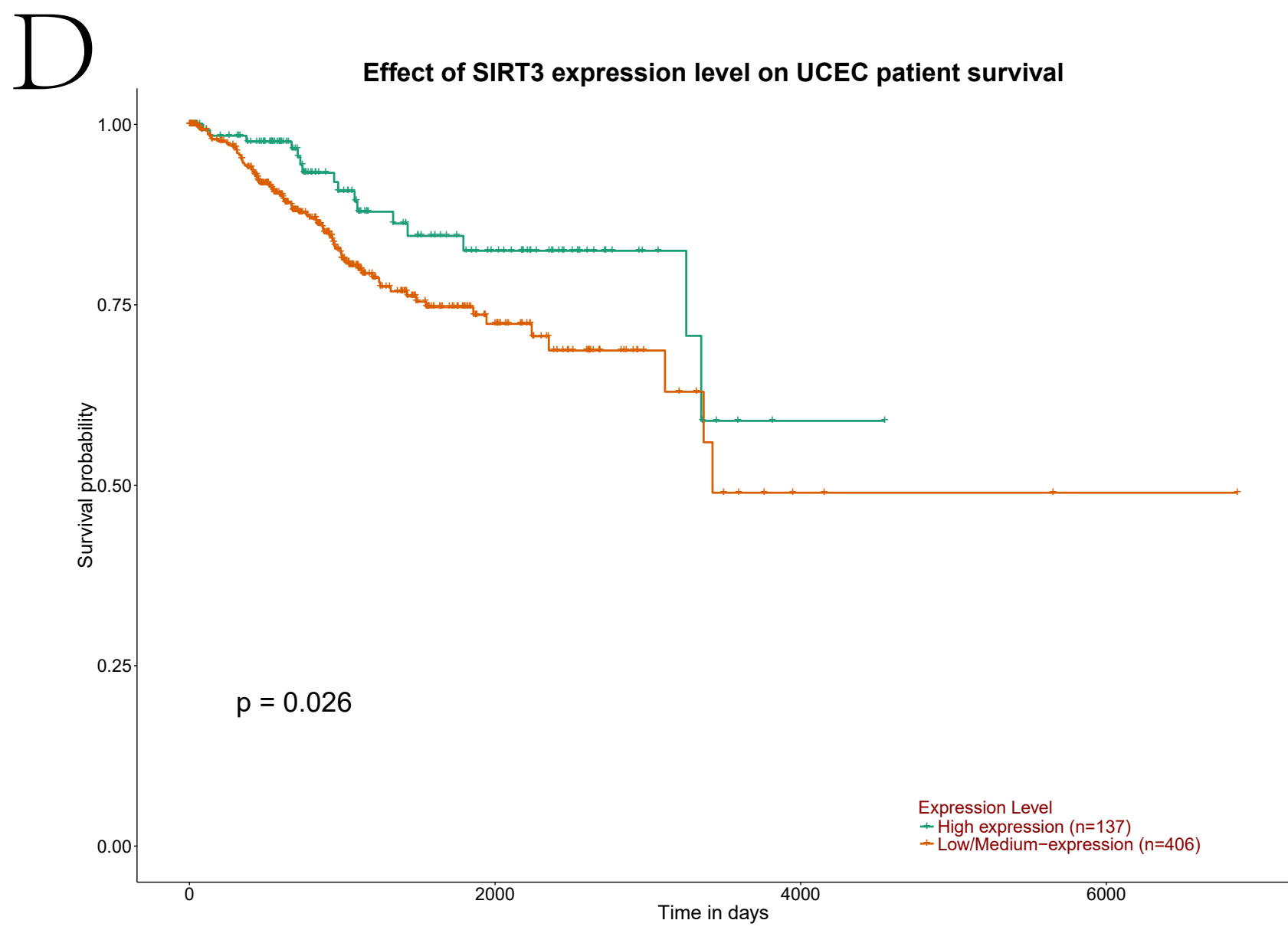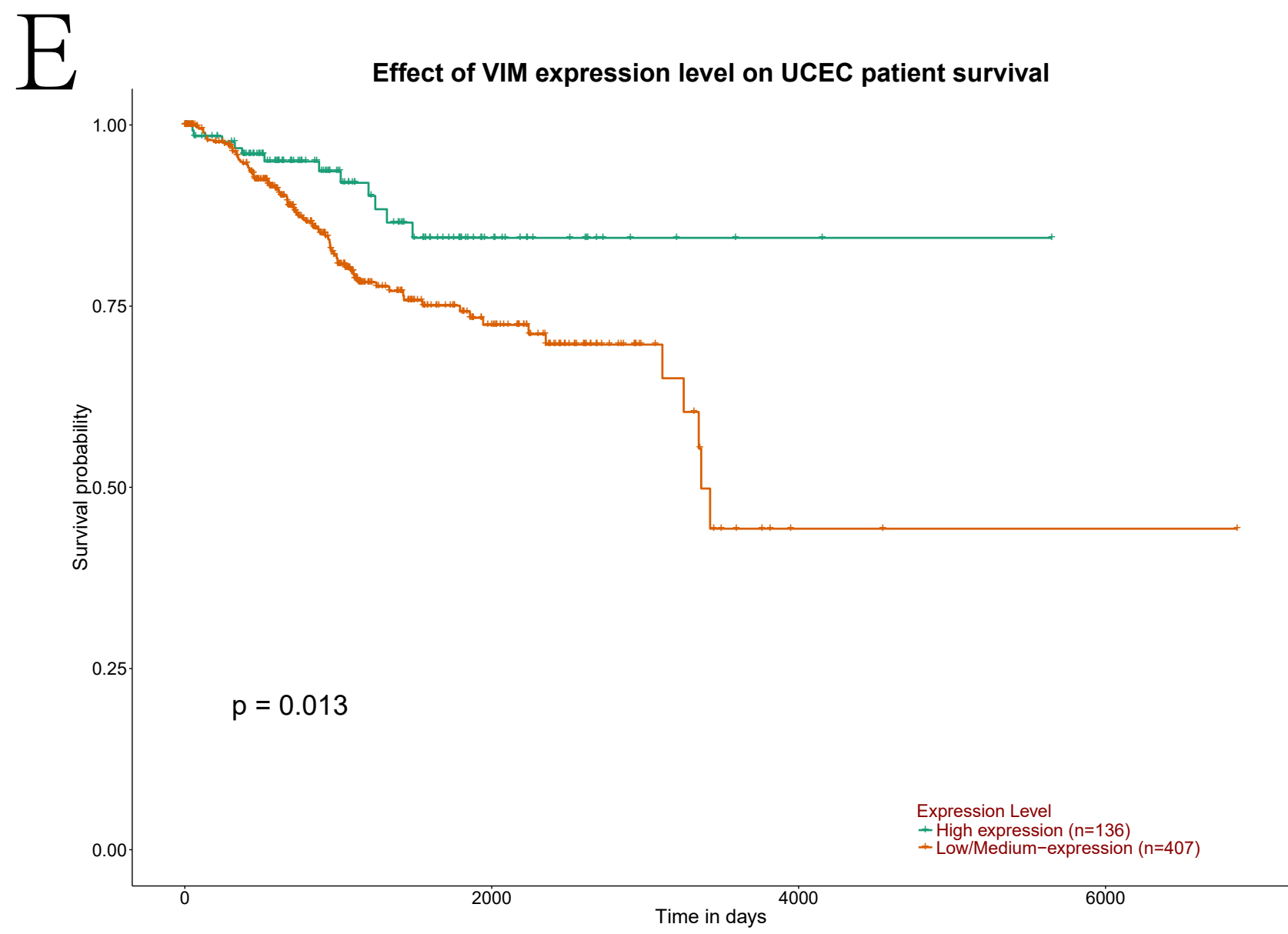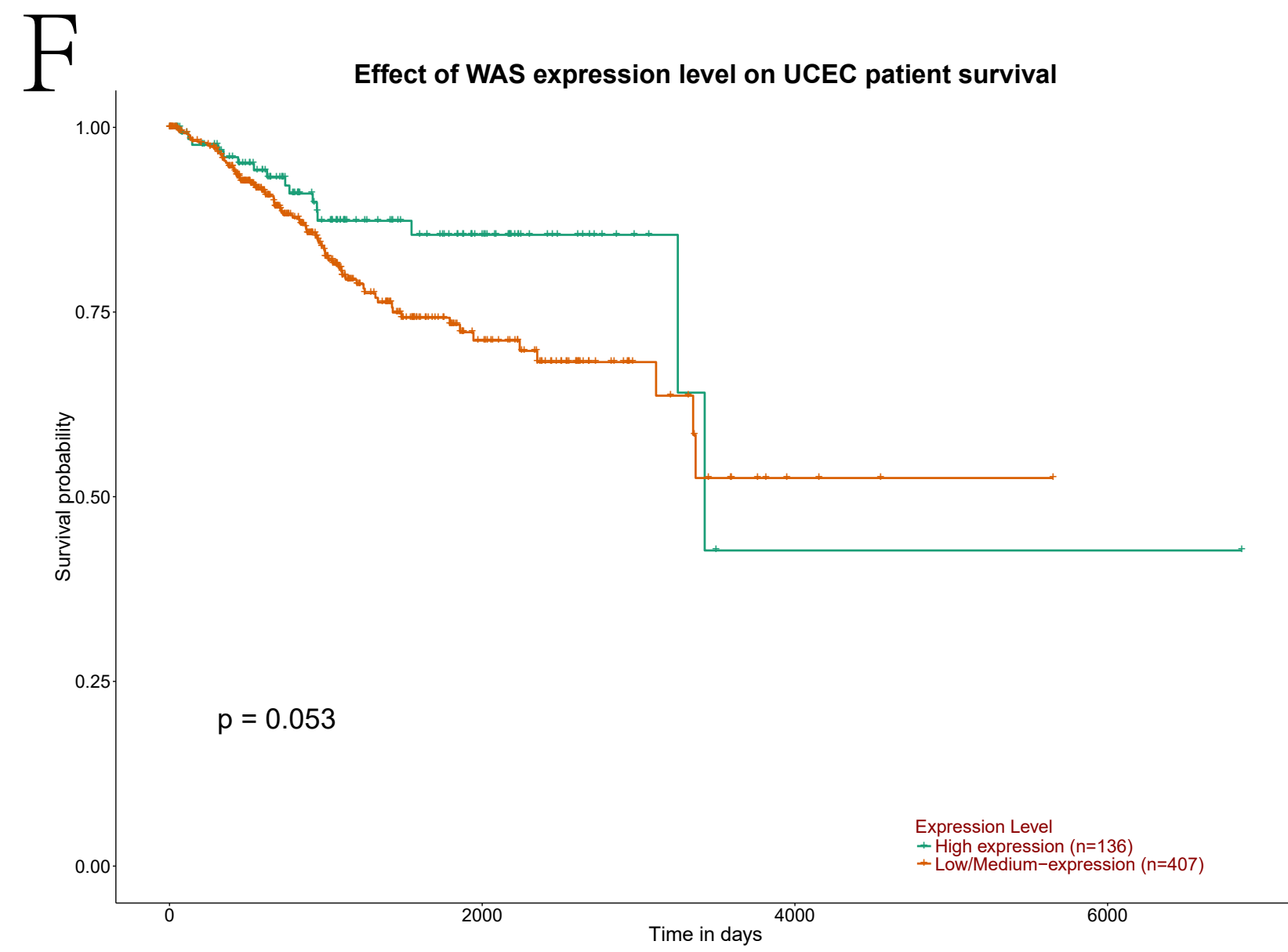

Supplement: Multimedia component 6 [file mmc6.pdf]
